# DNA Methylome Analysis of Saturated Aliphatic Aldehydes in Pulmonary Toxicity

**DOI:** 10.1038/s41598-018-28813-z

**Published:** 2018-07-12

**Authors:** Yoon Cho, Mi-Kyung Song, Tae Sung Kim, Jae-Chun Ryu

**Affiliations:** 1 Cellular and Molecular Toxicology Laboratory, Center for Environment, Health and Welfare Research, Korea Institute of Science and Technology (KIST), 5, Hwarang-ro 14-gil, Seongbuk-gu, Seoul 02792 Republic of Korea; 2 Department of Life Sciences, College of Life Sciences and Biotechnology, Korea University, 145 Anam-ro, Seongbuk-gu, Seoul 02841 Republic of Korea; 3 National Center for Efficacy evaluation for Respiratory disease product, Jeonbuk Department of Inhalation Research, Korea Institute of Toxicology, 30 Baehak1-gil, Jeongeup, Jeollabuk-do 53212 Republic of Korea; 4 Human and Environmental Toxicology, University of Science and Technology, 217, Gajeong-Ro, Yuseong-gu, Daejeon 34113 Republic of Korea

## Abstract

Recent studies have investigated the epigenetic effects of environmental exposure to chemicals on human health. The associations of DNA methylation, environmental exposure and human diseases have been widely demonstrated. However, the use of gene methylation patterns as a predictive biomarker for exposure to environmental toxicants is relatively poorly understood. Here, we focused on low-molecular-weight saturated aliphatic aldehydes (LSAAs), which are important environmental risk factors in humans as major indoor air pollutants. Based on DNA methylation profiling in gene promoter regions, we analysed DNA methylation profiles following exposure of A549 cells to seven LSAAs (propanal, butanal, pentanal, hexanal, heptanal, octanal, and nonanal) to identify LSAA-characterized methylated sites and target genes, as well as to investigate whether exposure to LSAAs contributes to inducing of pulmonary toxicity. Additionally, by integrating DNA methylation and mRNA expression profile analyses, we identified core anti-correlated target genes. Gene ontology analysis of these target genes revealed several key biological processes. These findings suggest that alterations in DNA methylation by exposure to LSAAs provide novel epigenetic biomarkers for risk assessments. This DNA methylation-mRNA approach also reveals potential new mechanistic insights into the epigenetic actions of pulmonary toxicity.

## Introduction

The field of epigenetics including DNA methylation, histone modification, and microRNAs is rapidly expanding, and these processes are associated with environmental exposure to pollutants. Recent studies have suggested that environmental pollutants can cause human diseases based on an epigenetic mechanism^1^. Epigenetics is essential for a wide range of biological processes including the regulation of gene expression, normal development, and cellular differentiation^2^. All epigenetic mechanisms play an important role in the pathogenesis of environmental diseases and cancers^3,4^.

Among the epigenetic mechanisms, there has been an increase in the number of DNA methylome studies investigating the effects of human health risk factors including environmental chemicals on disease. DNA methylation, which is the addition of methyl groups at the 5′ position on the pyrimidine ring of cytosine, is a crucial epigenetic modification of the genome^1^ and is important in regulating gene expression. Aberrant DNA methylation is associated with disease progression and human cancer^5,6^. To date, DNA methylation has been widely studied. Particularly, numerous studies demonstrating the influence of environmental toxicants on DNA methylation have been linked to human health^7^.

Various environmental toxicants such as air pollutants, toxic chemicals, and radiation that affect epigenetic processes result from the effects of acute or chronic exposure. Recent studies investigated whether multiple environmental exposure to polycyclic aromatic hydrocarbons, diesel exhaust particles, and particulate matter is a major risk factor for the development of environmental lung disease by epigenetic modifications^3^. However, the effects of exposure to major indoor air pollutants such as aldehydes on the respiratory system via DNA methylation remain unclear. Therefore, among environmental chemicals, we focused on low-molecular-weight saturated aliphatic aldehydes (LSAAs) for DNA methylome analysis. In previous studies, we investigated the genetic and epigenetic responses of LSAAs based on transcriptome and microRNA analyses^8,9^. Here, we performed epigenome-wide DNA methylome analysis in aldehyde-exposed A549 lung adenocarcinoma cells.

Aldehydes are categorized as volatile organic compounds, which are ubiquitous in indoor and outdoor air as common air pollutants^10^. They are emitted from diverse indoor sources such as carpets, floor coverings, paints, panels, and furniture^11^. Recognition of the health effects of indoor air pollutants has also increased. Although studies on the pulmonary effect of aldehydes have been conducted^12^, the data remain limited for several aldehydes such as formaldehyde and acetaldehyde. In this study, we investigated the pulmonary toxic effects of LSAAs including propanal, butanal, pentanal, hexanal, heptanal, octanal, and nonanal. To investigate the epigenetic DNA methylation biomarkers of aldehydes on the human respiratory system for health risk assessment, we performed DNA methylome analysis. Following DNA methylation profiling, we performed integrated analyses of the methylation and mRNA data in A549 cells exposed to aldehydes to identify correlations.

Taken together, these findings indicate the potential pulmonary toxic effects of aldehyde exposure. Such DNA methylation biomarkers also improve the understanding of the underlying mechanisms of epigenetic regulation associated with aldehyde exposure.

## Results

### Cytotoxicity of A549 cells exposed to aldehydes

To determine the optimal exposure concentrations, the cytotoxicities of seven aldehydes were evaluated in the MTT assay (Fig. S1). Based on the MTT assay data, the cell viability inhibitory concentrations (IC) of each aldehyde at 20% (IC_20_) were calculated and compared to those of the dimethyl sulfoxide (DMSO) vehicle control group. Each inhibitory concentration of the seven aldehydes is shown in Table 1.

**Table 1:** The 20% cell viability inhibitory concentrations (IC_20_) of seven aldehydes compared to vehicle control (DMSO) sample.

Aldehydes	IC_20_ (mM)
Propanal (C3)	2.5
Butanal (C4)	4.6
Pentanal (C5)	1.7
Hexanal (C6)	0.8
Heptanal (C7)	0.6
Octanal (C8)	0.58
Nonanal (C9)	0.44

### DNA methylation signatures following exposure to the seven aldehydes

**Figure 1:**
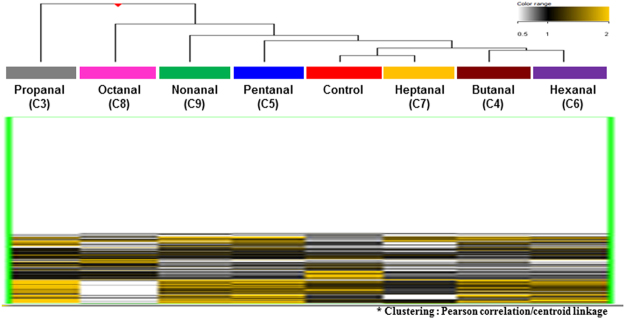
Total genome-wide profiling of DNA methylation of A549 cells exposed to the seven aldehydes compared to vehicle control group (DMSO). The heatmap shows the DNA methylation profiles of aldehydes exposed A549 cells based on hierarchical clustering (Yellow: hypermethylation; Black: hypomethylation).

To investigate the genome-wide promoter DNA methylation expression profiles in A549 cells exposed to aldehydes, we performed a human 2 × 400 k DNA methylation microarray (Agilent Technologies, Santa Clara, CA, USA). The 414,043 methylation probes resulted in the DNA methylation of A549 cells based on unsupervised hierarchical clustering analysis. We identified the total DNA methylation expression patterns in A549 cells exposed to the aldehydes (Fig. 1), and then examined the overall DNA methylation levels to identify aldehyde-specific epigenetic DNA methylation markers.

### Identification of characteristic aldehydes methylation markers

**Figure 2:**
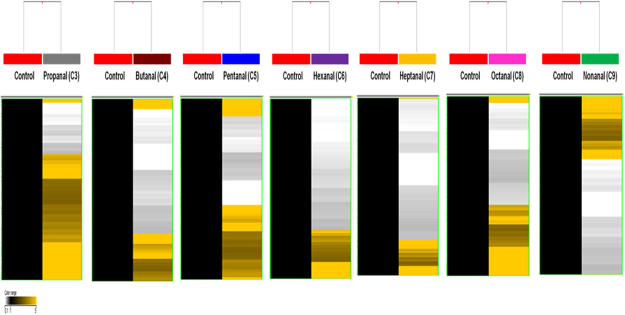
DNA methylation signatures of differentiated seven aldehydes compared to vehicle control group (DMSO) (Yellow: hypermethylation; Black: hypomethylation).

To determine whether exposure to aldehydes alters DNA methylation, we conducted DNA methylome analysis of seven aldehydes (C3–C9) using an *in vitro* model. First, we isolated methylated DNA using MeDIP from genomic DNA. Using this methylated DNA, a DNA methylation profile was acquired and analysed in the group exposed to the seven aldehydes and matched vehicle control group (Fig. 2, Table 2). In the propanal (C3) exposure group, we identified 4,345 genes that were differentially methylated (hyper-: 3,115 and hypo-: 1,230). In the butanal (C4) exposure group, 4,316 differentially methylated genes (hyper-: 1,584 and hypo-: 2,732) were identified. In the pentanal (C5) exposure group, 4,266 methylated genes (hyper-: 2,304 and hypo-: 1,962) were identified and 4,712 genes (hyper-: 1,572 and hypo-: 3,140) were methylated by hexanal (C6) exposure, and 3,598 target genes (hyper-: 897 and hypo-: 2,701) were identified following heptanal (C7) exposure. In the octanal (C8) exposure group, 3,822 methylated genes (hyper-: 1,611 and hypo-: 2,211) were identified, and 4,468 genes (hyper-: 1,898 and hypo-: 2,570) were methylated by nonanal (C9) exposure.

**Table 2:** Number of differentially methylated genes under exposure to seven aldehydes with 3.0-fold change and *p*-value < 0.05.

	Aldehydes						
	Propanal (C3)	Butanal (C4)	Pentanal (C5)	Hexanal (C6)	Heptanal (C7)	Octanal (C8)	Nonanal (C9)
Methylated site	9,741	10,405	9,931	12,164	9,023	8,891	11,316
Promoter region	4,896	4,957	4,859	5,608	4,170	4,263	5,268
Regulated gene	4,345	4,316	4,266	4,712	3,598	3,822	4,468

**Figure 3:**
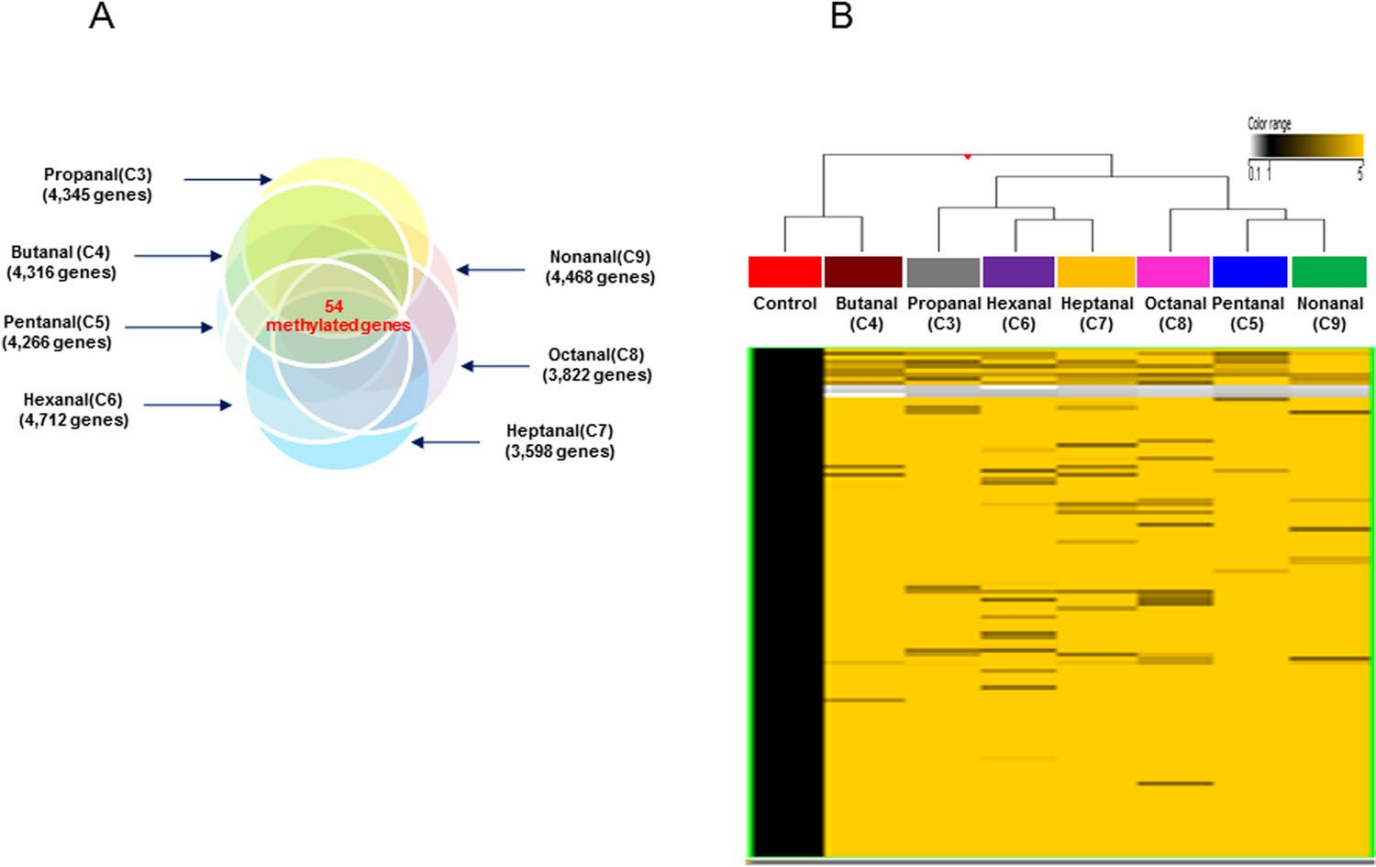
(**A**) Venn diagram shows the differentially methylated genes exposed under each aldehyde exposure. The intersection regions are the number of common differentially methylated genes following exposure to the seven aldehydes. (**B**) Hierarchical clustering of methylated genes that commonly altered DNA methylation in A549 cells exposed to the seven aldehydes with a fold-change ≥3.0-fold and *p*-value < 0.05 compared to the vehicle control group (DMSO) (Yellow: hypermethylation; Black: hypomethylation).

Among them, 54 genes showed common methylated expression patterns in the seven aldehydes exposure group (Fig. 3, Table 3). All methylated genes showed significant changes of 3.0-fold with p-values < 0.05.

**Table 3:** Commonly methylated genes of seven aldehydes exposed-A549 cell compared to vehicle control with 3.0-fold change and *p*-value < 0.05.

Gene Symbol	Gene name	Methylation degree (Fold-change)						
		C3	C4	C5	C6	C7	C8	C9
ZFHX2	zinc finger homeobox protein 2	12.98	3.32	10.00	8.15	3.33	11.36	13.54
TTTY5	testis-specific transcript, Y-linked 5	55.06	59.56	334.00	95.77	17.98	10.80	80.09
CCDC21;CEP85	centrosomal protein 85	66.14	49.39	52.05	164.01	134.69	129.86	86.00
GJA9-MYCBP	GJA9-MYCBP readthrough	8.75	3.55	6.45	6.10	3.69	8.61	12.25
PRKACB	protein kinase cAMP-activated catalytic subunit beta	823.47	78.87	1169.04	362.87	560.59	1057.51	1139.15
LPPR5	lipid phosphate phosphatase-related protein type 5 isoform 2	7.93	5.74	6.59	7.30	8.36	7.68	7.68
TDRKH	tudor and KH domain containing	6.99	10.40	7.49	7.88	6.52	9.72	13.72
OR10Z1	olfactory receptor family 10 subfamily Z member 1	3.17	3.88	3.62	4.89	3.68	5.29	6.37
NUF2	NUF2, NDC80 kinetochore complex component	8.99	27.81	37.15	15.14	4.05	5.39	6.90
KCNF1	potassium voltage-gated channel modifier subfamily F member 1	10.08	6.90	9.53	12.95	11.10	5.69	7.97
C2orf61; STPG4	sperm-tail PG-rich repeat containing 4	9.47	5.94	8.98	6.75	4.32	5.60	5.89
BMP10	bone morphogenetic protein 10	6.25	5.38	12.21	5.50	4.86	3.78	18.05
REG1A	regenerating family member 1 alpha	3.97	3.70	3.82	4.09	3.79	3.34	4.05
MIR128-1	microRNA 128-1	6.73	6.26	6.47	6.92	6.42	5.66	6.27
UBXN4	UBX domain protein 4	5.16	3.73	4.29	4.75	5.44	5.00	5.00
WDR75	WD repeat domain 75	6.02	5.01	6.58	6.02	6.12	14.02	7.04
THUMPD3	THUMP domain containing 3	5.45	9.95	5.95	5.45	5.54	5.78	3.27
COX17	COX17,cytochrome c oxidase copper chaperone	22.46	18.67	24.53	22.46	22.82	15.61	28.73
MFN1	mitofusin 1	12.89	13.22	10.36	3.21	4.97	3.23	27.85
NFXL1	nuclear transcription factor, X-box binding like 1	16.26	4.96	8.71	7.16	11.07	7.78	5.77
UGT2B10	UDP glucuronosyltransferase family 2 member B10	9.04	8.45	13.47	3.98	6.16	11.61	12.51
IGJ;JCHAIN	joining chain of multimeric IgA and IgM	10.61	9.91	15.07	4.68	7.22	13.63	14.68
HSD17B11	hydroxysteroid 17-beta dehydrogenase 11	13.20	8.61	10.78	5.08	4.48	6.00	7.62
SNCA	synuclein alpha	12.42	11.60	11.19	5.47	8.45	15.95	17.18
MIR583	microRNA 583	15.37	18.05	12.78	14.15	16.21	5.69	14.89
PCDHA9	protocadherin alpha 9	300.99	40.15	5.39	76.53	254.52	56.58	64.89
PCDHA10	protocadherin alpha 10	105.50	98.53	149.77	46.49	229.99	135.49	145.95
PHACTR1	phosphatase and actin regulator 1	21.89	16.73	9.00	6.72	5.74	15.07	8.65
HIST1H2BE	histone cluster 1 H2B family member e	42.08	21.83	91.80	60.34	13.59	3.23	41.49
TRIM27	tripartite motif containing 27	3.50	13.33	47.39	7.21	9.71	6.19	12.52
MRPS18A	mitochondrial ribosomal protein S18A	292.96	127.71	246.55	239.77	219.35	91.89	370.17
NMBR	neuromedin B receptor	15.41	14.39	21.87	6.79	10.49	17.90	21.32
LHFPL3	lipoma HMGIC fusion partner-like 3	27.40	51.13	17.34	61.21	16.23	25.98	19.18
PEX2	peroxisomal biogenesis factor 2	101.62	124.05	115.75	156.58	117.68	169.28	109.65
SPINK4	serine peptidase inhibitor, Kazal type 4	13.95	12.61	6.96	10.09	7.85	5.19	8.88
SLK	STE20 like kinase	3.89	6.52	25.12	4.00	3.71	3.27	35.02
PIK3C2A	phosphatidylinositol-4-phosphate 3-kinase catalytic subunit type 2 alpha	47.86	23.32	31.00	13.92	7.86	39.95	54.35
CD163L1	CD163 molecule like 1	8.33	16.73	25.48	3.28	7.12	9.91	14.92
PRH1-PRR4	PRH1-PRR4 readthrough	8.49	9.50	24.44	3.64	8.42	6.82	16.24
CCNA1	cyclin A1	15.92	9.51	13.60	5.87	3.17	12.83	15.04
CCNB1IP1	cyclin B1 interacting protein 1	3.45	16.63	16.41	3.20	9.03	9.13	11.86
C14orf104; DNAAF2	dynein axonemal assembly factor 2	12.81	8.80	7.24	4.70	3.62	3.95	5.63
C14orf166B; LRRC74A	leucine rich repeat containing 74 A	4.87	3.74	6.05	4.08	4.38	3.21	4.33
CSNK1A1P1	casein kinase 1 alpha 1 pseudogene 1	11.74	5.67	8.68	5.57	7.31	14.50	6.04
KIF7	kinesin family member 7	11.73	14.32	3.39	18.08	6.31	10.21	12.66
CHD3	chromodomain helicase DNA binding protein 3	28.65	23.21	35.98	15.25	14.61	15.85	10.57
PDE4A	phosphodiesterase 4A	7.78	11.77	7.15	5.46	9.41	8.80	8.16
ZNF525	zinc finger protein 525	11.18	7.40	24.49	4.92	7.61	17.13	15.78
C20orf191; NCOR1P1	nuclear receptor corepressor 1 pseudogene 1	8.70	10.62	9.91	13.40	10.07	14.49	16.16
MYT1	myelin transcription factor 1	16.18	7.61	13.72	5.89	3.82	3.87	6.82
AIRE	autoimmune regulator	4.90	4.56	19.72	5.04	4.68	4.12	4.57
ACOT9	acyl-CoA thioesterase 9	15.50	12.40	20.91	10.68	28.30	24.78	18.05
RPA4	replication protein A4	245.44	74.49	183.14	399.31	242.59	133.71	106.36
C9orf57	chromosome 9 open reading frame 57	0.29	0.23	0.28	0.08	0.26	0.23	0.23

### Integrated analysis of methylated DNA and mRNA expression profiles

**Figure 4:**
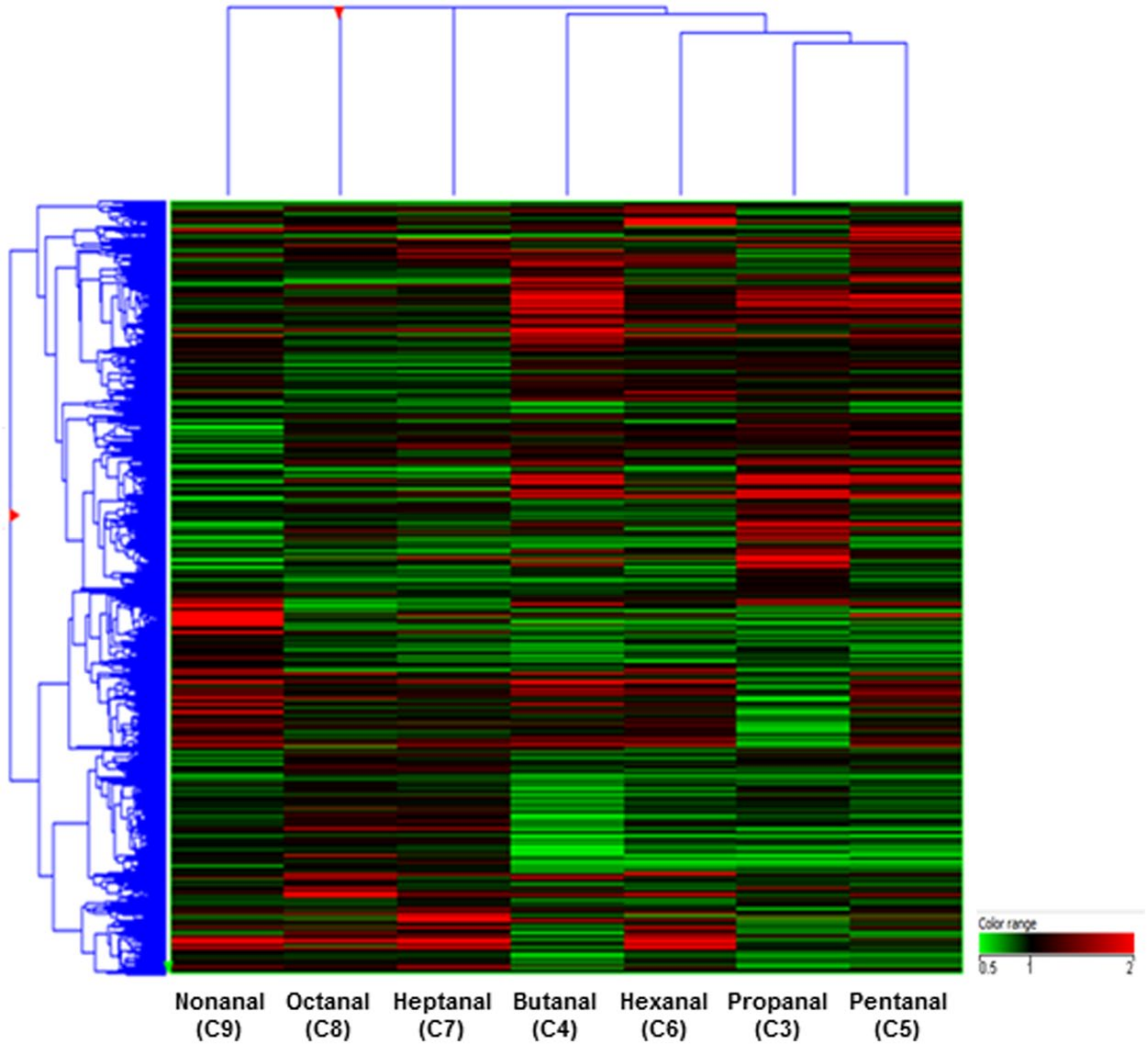
Heat map of differentially expressed genes in A549 cells exposed to the seven aldehydes using unsupervised hierarchical clustering. Colour intensity shows the differences in expression (Red: up-regulation; Green: down-regulation).

We also conducted integrated analysis of DNA methylation and mRNA expression. First, we conducted gene expression profiling of cells exposed to the seven aldehydes to identify differentially expressed genes (Fig. 4). The differentially expressed genes in the A549 cells exposed to aldehydes were detected using the Human Whole Genome Microarray 44 K array (GSE56005). Next, we performed an integrative analysis of DNA methylation and mRNA expression to investigate whether DNA methylation has a regulatory impact on gene expression following aldehyde exposure. We identified hyper-methylated and down-regulated genes and hypo-methylated and up-regulated ones in each of aldehyde exposure groups (Table 4). These genes were considered potential epigenetic biomarkers for determining the exposure of the LSAAs.

**Table 4:** Anti-correlated matching number of DNA methylation and mRNA expression by aldehydes exposure.

	Hyper-methylated vs. down-regulated	Hypo-methylated vs. up-regulated
Propanal (C3)	252	166
Butanal (C4)	248	483
Pentanal (C5)	157	242
Hexanal (C6)	123	338
Heptanal (C7)	72	269
Octanal (C8)	68	132
Nonanal (C9)	146	299

### GO and KEGG pathway analyses of candidate DNA methylation biomarkers of aldehydes

To investigate the related biological process and pathway of aldehyde-specific methylated genes, we performed GO and KEGG pathway analyses using DAVID bioinformatics resources. We analysed each aldehyde-matched DNA methylated genes. The common key biological processes were mainly involved in cell surface receptor linked signal transductions (GO:0007166) such as the G-protein coupled receptor (GPCR) signalling pathway (GO:0007186) and neurological system process (GO:0050877). KEGG pathway analysis revealed that aldehyde exposure was associated with neuroactive ligand-receptor interaction, calcium signalling pathway, and pathways in cancer. Although we identified overlapping similar biological processes and pathways for seven aldehydes, each aldehyde has distinct biological functions (Tables 5 and 6).

**Table 5:** Biological process of significant anti-correlated methylated genes by aldehydes exposure.

	GO Annotation (Biological process; BP)	Gene count	*p*-value (<0.05)
Propanal (C3)	G-protein coupled receptor protein signalling pathway	34	0.0123
	Response to hormone stimulus	18	9.64E-04
	Mitotic cell cycle	14	0.031
	Regulation of secretion	12	0.002
	Regulation of protein amino acid phosphorylation	10	0.007
Butanal (C4)	Cell surface receptor-linked signal transduction	84	0.006
	Neurological system process	56	0.017
	Cell motion	27	0.012
	Regulation of phosphorylation	24	0.049
	Response to hormone stimulus	23	0.008
	Protein kinase cascade	21	0.029
Pentanal (C5)	Cell surface receptor-linked signal transduction	46	0.017
	Regulation of cell proliferation	22	0.040
	Induction of apoptosis	13	0.012
	Reproductive developmental process	11	0.019
	Sensory organ development	10	0.021
Hexanal (C6)	Cell surface receptor-linked signal transduction	65	3.12E-05
	G-protein coupled receptor protein signaling pathway	44	8.25E-05
	Neurological system process	36	0.032
	Cell adhesion	29	8.21E-04
	Acute inflammatory response	7	0.017
Heptanal (C7)	Cell surface receptor-linked signal transduction	49	3.27E-04
	G-protein coupled receptor protein signaling pathway	34	3.80E-04
	Neurological system process	34	0.001
	Sensory perception	24	0.004
	Neuron differentiation	14	0.022
	Cell morphogenesis involved in differentiation	10	0.016
Octanal (C8)	Cell surface receptor-linked signal transduction	32	0.004
	Homeostatic process	14	0.047
	Cell motion	12	0.010
	Cell morphogenesis	9	0.032
	T cell activation	5	0.042
	Sensory perception of pain	3	0.043
Nonanal (C9)	Cell surface receptor-linked signal transduction	60	3.49E-04
	Neurological system process	40	0.003
	G-protein coupled receptor protein signaling pathway	36	0.009
	Sensory perception	26	0.028
	Response to hormone stimulus	14	0.040
	Regulation of cell migration	9	0.024

**Table 6:** KEGG pathway analysis of selected aldehydes specific anti-correlated methylated genes.

	KEGG pathway	Gene count	*p*-value (<0.05)
Propanal (C3)	Olfactory transduction	14	0.03
	cAMP signalling pathway	10	0.009
	Calcium signalling pathway	8	0.042
	Mineral absorption	4	0.049
Butanal (C4)	Pathways in cancer	22	0.030
	PI3K-Akt signalling pathway	21	0.016
	MAPK signalling pathway	18	0.007
	Focal adhesion	17	0.002
	Calcium signaling pathway	16	0.001
	Neurotrophin signaling pathway	12	0.003
Pentanal (C5)	Pathways in cancer	19	1.48E-04
	MAPK signalling pathway	13	0.002
	Calcium signalling pathway	12	2.79E-04
	Ras signalling pathway	11	0.006
	cAMP signalling pathway	10	0.008
Hexanal (C6)	Neuroactive ligand-receptor interaction	19	4.08E-06
	Pathways in cancer	15	0.016
	Ras signalling pathway	10	0.027
	Calcium signalling pathway	9	0.020
	Rap1 signalling pathway	9	0.046
Heptanal (C7)	Serotonergic synapse	7	0.007
	Vascular smooth muscle contraction	6	0.034
	Insulin secretion	5	0.040
Octanal (C8)	Neuroactive ligand-receptor interaction	9	0.008
	Calcium signalling pathway	6	0.038
Nonanal (C9)	Pathways in cancer	17	0.002
	Neuroactive ligand-receptor interaction	13	0.004
	cAMP signalling pathway	12	9.25E-04
	Calcium signalling pathway	11	0.002
	MAPK signalling pathway	11	0.018
	Serotonergic synapse	8	0.004

## Discussion

Saturated aliphatic aldehydes are widely used in perfumes, essential oils, and flavoring^13^. They are also considered as indoor and outdoor air pollutants^14^. Human exposure to aldehydes occurs mainly by inhalation. Therefore, several studies have monitored the levels of aldehydes in the environment and reported the potential adverse health effects on humans^15^. Additionally, previous studies demonstrated that aldehydes may contribute to the development of various disease including pulmonary inflammatory diseases such as tracheitis, bronchitis, and bronchiolitis^16^. Among the aldehydes, formaldehyde and acetaldehyde are the most widely studied in compaison with other aldehydes. Little information is available regarding the toxicity of the other aldehydes, although numerous studies have conducted aldehyde measurement in indoor environments. Therefore, we investigated the relationship between exposure to other aldehydes and potential adverse health effects associated with pulmonary toxicity.

Epigenetic studies are important for examining the association between environmental exposure and health outcomes and have been utilized for exposure assessment. However, few studies have investigated the association between environmental factors including aldehydes and genome-wide epigenome in humans. Therefore, we focused on epigenetic alterations of saturated aliphatic aldehydes and aldehyde-specific DNA methylation changes as potential biomarkers.

We analysed the DNA methylation patterns of the seven saturated aliphatic aldehydes from propanal (C3) to nonanal (C9) in A549 cells. To identify the critical DNA methylation-based biomarkers of aldehydes, we investigated the correlation between DNA methylation profiles and mRNA expression using microarrays. Microarray technology is a powerful tool for evaluating environmental chemicals on human health, providing valuable genomic information for identifying biomarkers related to occupational exposure and disease prognosis^17–19^.

Using a DNA methylation array, we identified methylated genes specific for each aldehyde as well as commonly methylated genes among the seven aldehydes at the IC_20_, which shows low cytotoxicity and is useful for identifying genes. A total of 54 genes were commonly methylated compared to in control cells, with a greater than 3.0-fold change cut-off and *p*-value < 0.05. These methylation alterations were considered as important potential epigenetic-based biomarkers, which can be used to assess cases of aldehyde exposure and predict pulmonary toxicity induced by aldehyde exposure.

Aberrant DNA methylation has been associated with numerous human diseases, cancer, and environmental exposure^20–22^. Furthermore, hyper/hypo-methylated genes can modify the function of genes and regulate gene expression. Generally, DNA hypermethylation leads to down-regulation of genes and hypomethylation allows for up-regulation of genes. The inverse correlation between DNA methylation and mRNA expression plays critical roles and can serve as potential epigenetic biomarkers. Therefore, we also investigated the correlation between DNA methylation and mRNA expression to identify critical epigenetic biomarkers of aldehydes. In each aldehyde exposure group, we identified numerous anti-correlated methylated genes (Table 4). To further investigate the biological relevance of these genes, we performed functional analyses such as GO and KEGG pathway analyses using the DAVID bioinformatics tool (https://david.ncifcrf.gov/). Furthermore, GO terms and KEGG pathways are important indicators for elucidating the biological functions of genes^23^.

First, GO analysis showed that aldehyde-associated epigenetic biomarkers were associated with cell surface receptor-linked signal transduction, cell adhesion, inflammatory response, neurological system process, apoptosis, and the GPCR signalling pathway. Among the biological processes, cell surface receptor-linked signal transduction and the GPCR signalling pathway were mainly involved in biological processes related to aldehyde exposure. Further studies are needed to determine the molecular mechanisms involved in the effects of aldehyde exposure on the GPCR signalling pathway. Next, we conducted KEGG pathway analysis to identify detailed information on the key biological pathways relevant to aldehyde exposure. The calcium signalling pathway, MAPK signalling pathway, and cancer pathways were linked to the biological significance of aldehydes (Table 5). Among the related biological pathways, we previously demonstrated that exposure to butanal and octanal induced MAPK cascades in A549 cells^24,25^. The MAPK cascades are central signalling pathways in cellular processes that mediate a variety of physiological and pathological functions^26^. Therefore, further studies are required to determine whether aldehydes induce cellular metabolism via MAPK cascades.

Taken together, these findings demonstrate that DNA methylation-based epigenetic biomarkers of aldehydes in the human respiratory system can be used for exposure assessment and predicting pulmonary toxicity. Our results also improve the understanding of the underlying mechanisms of aldehyde exposure, which may be useful for determining the developmental toxicological mechanisms of aldehyde-induced environmental lung disease.

## Materials and Methods

### Chemicals and reagents

Aldehydes (propanal, butanal, pentanal, hexanal, heptanal, octanal, nonanal), dimethyl sulfoxide (DMSO), and 3-(4,5-dimethylthaizol-2-yl)-2,5-diphenyltetrazolium bromide (MTT) were purchased from Sigma–Aldrich (St. Louis, MO, USA). The following cell culture media and supplemented buffer solutions were purchased from GIBCO™ (Grand Island, NY, USA): Roswell Park Memorial Institute (RPMI) 1640, Dulbecco’s phosphate buffered saline, foetal bovine serum, and antibiotics (penicillin and streptomycin). All chemicals used in this study were analytical grade or the highest grade available.

### Cell culture

The human lung adenocarcinoma epithelial cell line (A549) was obtained from the Korea Cell Line Bank (Seoul, Korea) and grown in RPMI1640(Gibco) supplemented with 10% foetal bovine serum, sodium bicarbonate, HEPES, and penicillin under a humidified atmosphere of 5% CO_2_ and 95% air at 37 °C. The culture medium was replaced every 2 or 3 days.

### Chemical treatment

A549 cells were treated with each chemical compound at 1.0% of final solvent (DMSO) concentration in multi-well plates. Because of the volatility of the compounds, the plates were sealed with sealing films after chemical treatment. A549 cells were seeded into a 24-well plate at a density of 7.0 × 10^4^ cells/mL per well in 500 μL of media for the cytotoxicity assay and seeded into a 6-well plate at a density of 25.0 × 10^4^ cells/mL for RNA and genomic DNA extraction. After incubation for 24 h, the cells were treated with the seven aldehydes for 48 h.

### Determination of cell viability

To determine the cell viability and cytotoxic effects of the aldehydes, an MTT cell proliferation assay was performed as described previously by Mosmann^27^.

### Preparation of genomic DNA

Genomic DNA was extracted from A549 cells exposed to the seven aldehydes using the QIAamp DNA Mini Kit (Qiagen, Hilden, Germany) according to manufacturer’s instructions. The amount of each genomic DNA was measured using a NanoDrop ND 1000 spectrophotometer (NanoDrop Technologies, Inc., Wilmington, DE, USA). Only clear samples without a substantial smear were considered suitable for use and their quality was checked by electrophoresis in a 1.5% agarose gel in 1X TAE buffer (4.8 g of Tris, 1.14 mL of acetic acid, 2 mL of 0.5 M EDTA at pH 8.0, and ethidium bromide) at a constant 100 V for 15 min.

### Fragmentation of genomic DNA

Genomic DNA was randomly fragmented using a Sonic Dismembrator 550 (Fisher Scientific, USA) as described previously by Song *et al*.^28^. Briefly, 5 μg of DNA in 0.2 mL was placed into 1.5-mL tubes immersed in ice and sonicated for various times (10, 20, and 40 s) with a model Sonic Dismembrator 550 (Fischer Scientific, USA) set at 30 W, 1/8-inch horn, and 5% maximum output placed in the centre of the DNA solution at a depth of 5 mm. The size range of the fragmented DNA was evaluated by agarose gel electrophoresis and ethidium bromide staining using DNA size markers 500–10,000 base pairs in size.

### Immunoprecipitation of methylated DNA (MeDIP)

MeDIP was performed using the MethylMiner Methylated DNA Enrichment Kit (Invitrogen, Carlsbad, CA, USA) following the manufacturer’s instructions. One microgram of fragmented DNA and 3 μg of untreated control DNA (Input) were used for downstream quality procedures and labelling. First, 7 μL of MBD-Biotin Protein was coupled to 10 μL of Dynabeads M-280 Streptavidin according to the manufacturer’s instructions.

The MBD-magnetic bead conjugates were washed three times and resuspended in 1 volume of 1X bind/ wash buffer. The capture reaction was conducted by adding of 1 μg sonicated DNA to the MBD magnetic beads on a rotating mixer for 1 h at room temperature (RT). All capture reactions were performed in duplicate. Next, the beads were washed three times with 1X bind/wash buffer. The methylated DNA was eluted as a single fraction with a high-salt elution buffer (2,000 mM NaCl). Subsequently, each fraction was concentrated by ethanol precipitation using 1 μL glycogen (20 μg/μL), 1/10th volume of 3 M sodium acetate (pH 5.2), and two volumes of 100% ethanol, and then resuspended in 60 μL of DNase-free water. The eluted MeDIP pellet was stored at −20 °C for long-term storage.

### Whole genome amplification (WGA)

After purification and quantification, MeDIP and Input DNA were amplified using the GenomePlex® Complete Whole Genome Amplification (WGA2) Kit (Sigma-Aldrich) according to the modified manufacturer’s instructions. Two points were modified from the supplier’s recommendations: first, the initial DNA fragmentation step was omitted because sonicated DNA was used. Second, 20 ng of IP and Input DNA were used rather than 10 ng. The reactions were cleaned by purification with a column-based technique (QIAquick PCR cleanup columns, Qiagen). Each amplified DNA was quantified using the NanoDrop ND 1000 spectrophotometer. The quality and success of WGA were assessed by agarose gel electrophoresis to evaluate the conservation of size distribution after WGA.

### Analysis of promoter DNA methylation and gene expression profile

DNA methylation analysis was conducted on the WGA samples using the Human promoter 2 × 400 K methylation array (Agilent Technologies, Santa Clara, CA, USA). Briefly, 1 μg of amplified DNA and methylated IP samples were annealed with 5 μL of random primer for 3 min at 95 °C and then placed on ice for 5 min. WGA samples were labelled with Cy5 (red) for fully methylated DNA or Cy3 (green) for fragmented DNA using a randomly primed Klenow polymerase reaction using the Agilent Genomic DNA Enzymatic Labeling Kit (Agilent Technologies) according to the manufacturer’s instructions. After purification, labelled samples were resuspended with blocking reagent and hybridization buffer, followed by boiling for 3 min at 95 °C and incubated for 30 min at 37 °C, and then hybridized to arrays for 40 h at 67 °C while rotating at 800 RPM in a hybridization oven for 3 min. After washing, the slide was dried and hybridization images on the slides were scanned by the Agilent C scanner and analysed using Agilent Feature Extraction software (v10.7.3.1). All data normalization and selection of fold-changed probes were performed using GeneSpringGX 7.3 (Agilent Technologies). Probes showing a greater than 3.0-fold difference in the ratio between the test and control samples were selected and considered as differentially methylated probes.

Gene expression profiling of A549 cells exposed to aldehydes was conducted using the 44 k Whole Human Genome Microarray (Agilent Technologies) as described previously by Song *et al*.^25^. The data were normalized by dividing the average normalized treated signal intensity by the average normalized control intensity.

### Comparative analysis of DNA methylation and mRNA

Comparative analysis of methylation and mRNA data was performed to identify the anti-correlation associated with exposure to aldehydes using GeneSpring GX.

### Gene Ontology (GO) category analysis

To determine the functional categories of methylated target genes, the DAVID Gene Functional Classification Tool (https://david.ncifcrf.gov/) was used. Using the DAVID tool, pathway analyses were also conducted based on the Kyoto Encyclopedia of Genes and Genomes (KEGG) Pathways, which was linked to the KEGG pathway map.

### Statistical analysis

The differences between control and exposure subjects were evaluated using the unpaired *t*-test. The *p*-value criterion was set at *p*-value < 0.05 as the level of statistical significance.

### Data availability

The datasets generated during the current study are available from the corresponding author upon reasonable request.

## Electronic supplementary material

Supplementary Information
