# Supplementary material for: DNA Methylome Analysis of Saturated Aliphatic Aldehydes in Pulmonary Toxicity
Source: Sci Rep. 2018 Jul 12;8:10497. doi: 10.1038/s41598-018-28813-z (PMC6043580; doi:10.1038/s41598-018-28813-z)
Supplement: Supplementary file 1 — Supplementary Information [file 41598_2018_28813_MOESM1_ESM.pdf]

Supplementary Information

DNA Methylome Analysis of Saturated Aliphatic Aldehydes in Pulmonary Toxicity

Yoon Cho[1,2], Mi-Kyung Song[3], Tae Sung Kim[2] and Jae-Chun Ryu[1,4*]

[1] Cellular and Molecular Toxicology Laboratory, Center for Environment, Health and Welfare Research, Korea Institute of Science and Technology (KIST), 5, Hwarang-ro 14-gil, Seongbuk-gu, Seoul, 02792, Republic of Korea

[2] Department of Life Sciences, College of Life Sciences and Biotechnology, Korea University, 145 Anam-ro, Seongbuk-gu, Seoul, 02841, Republic of Korea

[3] National Center for Efficacy evaluation for Respiratory disease product, Jeonbuk Department of Inhalation Research, Korea Institute of Toxicology, 30 Baehak1-gil, Jeongeup, Jeollabuk-do, 53212, Republic of Korea

[4] Human and Environmental Toxicology, University of Science and Technology, 217, Gajeong-Ro, Yuseong-gu, Daejeon, 34113 Republic of Korea

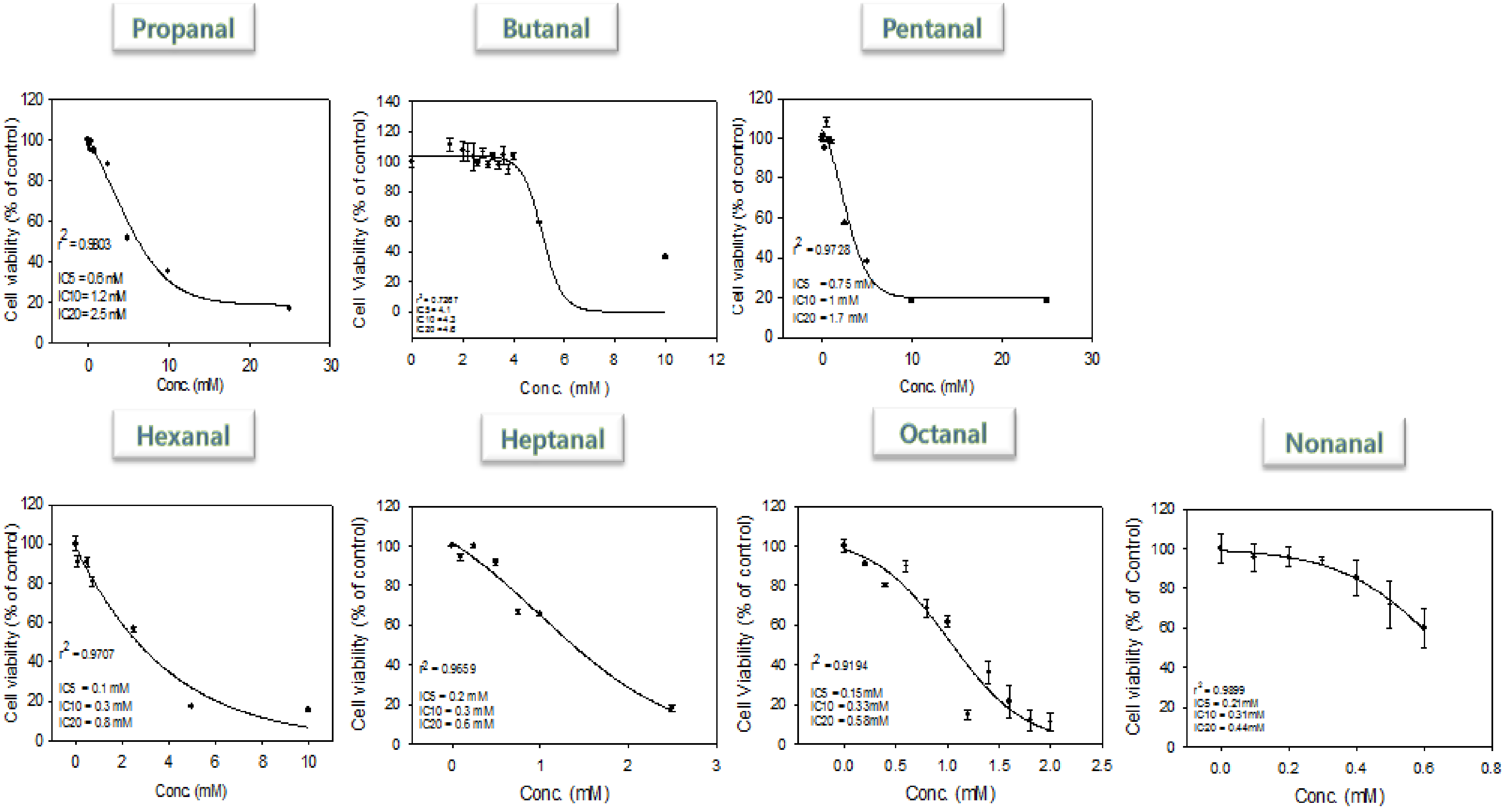

Figure 1. Cell viability of A549 cells exposed to the seven aldehydes using MTT assay.
